# Informational connectivity: identifying synchronized discriminability of multi-voxel patterns across the brain

**DOI:** 10.3389/fnhum.2013.00015

**Published:** 2013-02-07

**Authors:** Marc N. Coutanche, Sharon L. Thompson-Schill

**Affiliations:** Department of Psychology, University of PennsylvaniaPhiladelphia, PA, USA

**Keywords:** MVPA, fMRI, method, multivariate, networks, connectivity, pattern discriminability

## Abstract

The fluctuations in a brain region's activation levels over a functional magnetic resonance imaging (fMRI) time-course are used in functional connectivity (FC) to identify networks with synchronous responses. It is increasingly recognized that multi-voxel activity patterns contain information that cannot be extracted from univariate activation levels. Here we present a novel analysis method that quantifies regions' synchrony in multi-voxel activity pattern discriminability, rather than univariate activation, across a timeseries. We introduce a measure of multi-voxel pattern discriminability at each time-point, which is then used to identify regions that share synchronous time-courses of condition-specific multi-voxel information. This method has the sensitivity and access to distributed information that multi-voxel pattern analysis enjoys, allowing it to be applied to data from conditions not separable by univariate responses. We demonstrate this by analyzing data collected while people viewed four different types of man-made objects (typically not separable by univariate analyses) using both FC and informational connectivity (IC) methods. IC reveals networks of object-processing regions that are not detectable using FC. The IC results support prior findings and hypotheses about object processing. This new method allows investigators to ask questions that are not addressable through typical FC, just as multi-voxel pattern analysis (MVPA) has added new research avenues to those addressable with the general linear model (GLM).

## Introduction

The enormous wealth of data generated by functional magnetic resonance imaging (fMRI) has driven the continual development of new analytical methods to understand the brain's functions and processes. For many years, a predominant analysis approach has applied the general linear model (GLM) to compare blood oxygenation level dependent (BOLD) univariate activation levels across conditions, regions and subject groups (Friston et al., 1994). The last 10 years, however, have seen increased recognition within the fMRI community that information can also be encoded in the activity patterns of populations of voxels. A multitude of studies have now successfully employed multi-voxel pattern analysis (MVPA) techniques to decode information contained within multi-voxel activity patterns (Haynes and Rees, 2006; Norman et al., 2006; O'Toole et al., 2007). Many such studies have reported that their conditions of interest could not be distinguished by the mean voxel response differences that are assessed in a univariate GLM approach (e.g., Haxby et al., 2001).

In this study, we introduce an analysis method that combines MVPA's access to distributed encoding, with connectivity analyses. Functional connectivity (FC) techniques measure the degree of response-level synchrony between different brain regions or voxels (Biswal et al., 1995). The particular measures used to index connectivity (during rest or while performing a task) vary with different approaches (e.g., Friston et al., 1997), but a frequent goal is to identify regions with response levels that fluctuate in a synchronized manner. Just as univariate analyses have led to numerous findings, GLM's cousin—the analysis of *fluctuating* univariate responses of voxels or regions (FC)—has led to results in a wide spectrum of research fields. In this paper, we introduce a method–Informational Connectivity (IC)—that could analogously be considered a cousin of MVPA.

As discussed above, multi-voxel pattern investigations have revealed that one voxel's response magnitude is frequently insensitive to information encoded across a pattern of voxels. Instead of comparing the magnitude of activation levels, multi-voxel analyses frequently employ a machine learning classifier to assess the multivariate discriminability of conditions. While GLM investigations look to increased or decreased response levels as an indication of relevant neural activity, studies using MVPA often consider the successful separation of conditions as being an indicator of relevant neural information. In this paper, we introduce a method that quantifies the discriminability of multi-voxel patterns in a seed region and identifies regions of the brain that show synchronized discriminability over time.

**Figure 1:**
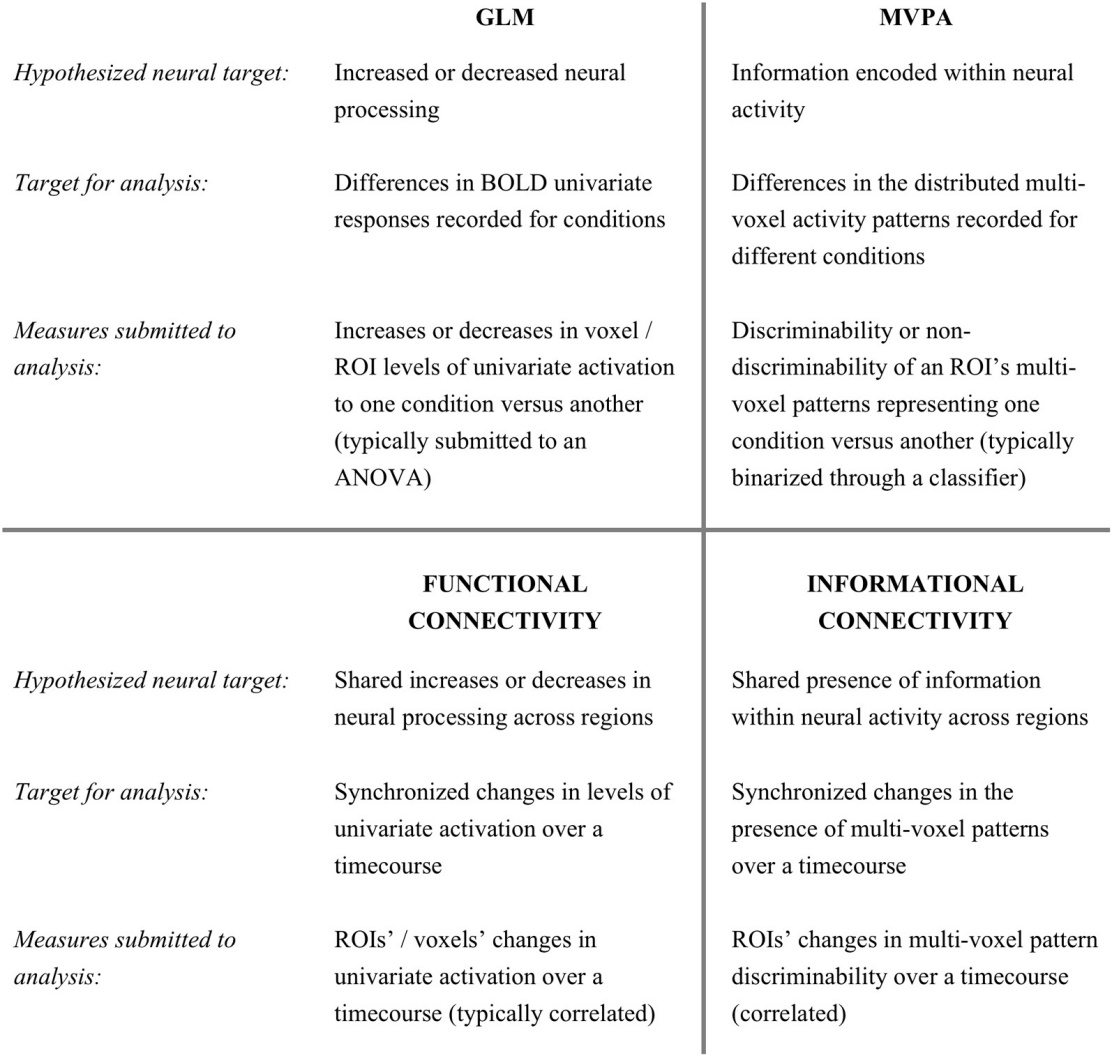
**The relationship between Informational Connectivity and other fMRI measures**.

Whereas FC is frequently applied to measure connectivity between a seed and individual brain voxels, it is (by definition) not possible to measure multi-voxel patterns in single voxels. Instead, we quantify how well a condition can be discriminated from other conditions in the *multi*-voxel patterns at each time-point in a scanning session. We measure the time-course of discriminability for a seed region and for 3-dimensional spheres (“searchlights”) placed at every location in the brain. We correlate the seed region's discriminability time-course with the equivalent time-course of each searchlight: measuring the simultaneous ebb and flow of multi-voxel distributed information across regions (compared to FC in Figure 1).

Since the conference presentation of an earlier version of this work (Coutanche and Thompson-Schill, 2011), Chiu et al. (2012) have employed a FC framework to identify voxels that vary in univariate responses for two cognitive states that were identified by a multivariate classifier in a region-of-interest (ROI). Our approach contrasts with this by identifying regions that have synchronized discriminability of multi-voxel information (rather than changing univariate activation). This makes our technique available for examining conditions that are not accompanied by differing univariate responses. Multivariate techniques have previously been applied in alternative connectivity approaches (such as the application of information-theoretical measures; Chai et al., 2009; Lizier et al., 2011). Our approach contrasts with prior work that has applied multivariate analyses to FC results (e.g., Welchew et al., 2005), by employing its own metric (instead of analyzing univariate change) to track multi-voxel pattern discriminability, building on the success of MVPA at detecting information inaccessible to univariate measures. This distinction is analogous to the difference between using MVPA and applying multivariate analyses to a GLM map. Although both approaches might yield interesting results, MVPA is sensitive to the condition-relevant distributed information that is coded within populations of voxels.

Here, we describe our method by example and examine its effectiveness by applying it to a classic dataset from Haxby et al. (2001); later analyzed in Hanson et al. (2004), O'Toole et al. (2005), and Raizada and Connolly (2012). For simplicity, and to test our technique's sensitivity to conditions that are distinguishable by potentially subtle differences in activity patterns, we restrict our analyses to time-points associated with presentations of four man-made object categories. We select six seed regions and identify brain areas that are informationally connected to each. We compare these results to a conventional FC analysis. The possible differences between these two methods include IC revealing: a subset of FC (selectivity), a superset of FC (sensitivity), a different set of regions, or no regions. We predicted that IC would identify more areas of cortex than FC, based on findings that multivariate decoding can detect information that a typical GLM cannot (Haxby et al., 2001) and a recent direct comparison of MVPA and GLM showing that MVPA can identify more areas of relevant cortex (Jimura and Poldrack, 2012). IC has MVPA's sensitivity and access to distributed information that is not obtainable from univariate responses. The larger IC networks might include the FC regions (i.e., a superset) or there may be little overlap. In their comparison of MVPA and GLM results, Jimura and Poldrack (2012) noted that a “conjunction of the two analyses revealed relatively small commonality in significant results across the brain” (p. 549), leading us to predict that informational and functional networks may be largely distinct. One consequence of this predition is an expectation that some regions will be identified based on common univariate synchrony (FC) but not multivariate synchrony (IC). This hypothesis is supported by prior findings that univariate differences can sometimes identify regions that are not identified from MVPA (Quamme et al., 2010; Jimura and Poldrack, 2012).

## Materials and methods

### Stimuli and experimental design

Full experimental details are available from the original manuscript employing this data (Haxby et al., 2001), but the relevant details are as follows. Participants were presented with 24-s blocks (separated by 12 s of rest) of gray-scale photographic images belonging to one of eight categories: faces, houses, cats, scrambled images, bottles, chairs, shoes, and scissors. For these analyses we focused on the latter four categories (all man-made objects). Within blocks, stimuli were presented for 500 ms with an interstimulus interval of 1500 ms. Participants identified object repeats (1-back) with a button-press. One block of every category appeared in each of 12 runs (excepting one participant where 11 runs were available). Analyses were performed on data for all runs from the five participants with anatomical T1-images and functional datasets available. The condition-labels for the time-points were shifted by two TRs for the multi-voxel pattern and IC analyses to account for the hemodynamic delay, giving nine TRs for each block and 108 for each condition across the experiment.

### Imaging preprocessing

Hemodynamic changes were recorded with gradient echo echo-planar imaging with a 3T scanner [repetition time (TR) = 2.5 s, forty 3.5 mm thick sagittal slices, TE = 30 ms, flip angle = 90; Haxby et al., 2001]. The functional data were slice-time corrected, motion-corrected, aligned to the subject's anatomical image and detrended with a second order polynomial. The anatomical image and functional data were transformed into standardized Talairach space with unchanged voxel resolution (3.5 × 3.75 × 3.75 mm for functional data). For the IC analyses, the effects of motion and global signal were removed from the data by modeling six motion parameters (pitch, roll, yaw, x, y, z) and mean white matter signal, and then using the residuals for subsequent analyses. This is equivalent to including motion and white matter signal as covariates in a FC model. The white matter signal was extracted using SPM8's segmentation procedure, which classifies voxels into gray matter, white matter and cerebrospinal fluid based on image intensity and prior probabilities of the distribution of tissue types. A threshold of 0.75 was employed to select white matter voxels. The Analysis of Functional NeuroImages (AFNI) software package was used for preprocessing and relevant univariate analyses (Cox, 1996). Prior to MVPA and IC analyses, each voxel's task and rest data were *z*-scored within each run; normalizing the run's time-series to have a mean of zero and unit variance.

### Seed regions

We examined IC and FC for six empirically determined seed regions: two regions identified by both an MVPA searchlight and GLM group map; two regions found from the MVPA searchlight but not the GLM; two regions found in the GLM but not the searchlight. To create the relevant group MVPA searchlight map, each individual's dataset was submitted to a 4-way correlation-based classifier (a popular classification approach) to separate activity patterns from the four types of man-made objects. We implemented a roaming searchlight analysis (Kriegeskorte et al., 2006), where a spherical volume (3-voxel radius) is centered on each brain voxel in turn and an analysis (in this case, classification) is conducted using data from the voxels included within the searchlight volume. For each searchlight, a leave-one-run-out cross-validation procedure trained on 11 runs and tested on the twelfth. Each testing TR's vector of activity values was correlated with the mean activity pattern for each of the four conditions in the training set. The condition that was most strongly correlated with the testing time-point determined the classifier's prediction for that TR. Classifier performance was calculated as the proportion of correctly predicted time-points (chance = 25%). The classification accuracy from each searchlight was allocated to its central voxel for mapping purposes. Individual searchlight maps were smoothed (9 mm Full-Width at Half Maximum; FWHM) and subjected to a one-way group *t*-test for performance above chance. As this was performed purely to identify seeds, we adopted a liberal threshold of *p* < 0.005 and cluster size of at least five voxels.

To create a group GLM map, each individual's dataset was submitted to a typical univariate analysis with six motion parameters as covariates. As the above searchlight analysis attempted to distinguish the four man-made objects, we ran a similar analysis with the GLM: running six pairwise comparisons, smoothing each individual's pairwise maps (9 mm FWHM) and submitting the maps for each comparison to a group analysis. The six group maps were then thresholded at *p* < 0.005 and a union of the six maps was created. A 5-voxel cluster threshold was then applied. Relatively few voxels survived even this liberal threshold, as expected from prior literature showing that object identity is typically not identifiable from univariate differences (Haxby et al., 2001).

The six seeds were created by selecting the central voxels of the two largest cluster volumes found only in the searchlight map, the two largest found only in the GLM map (although as discussed above, this was at a sub-significant level), and the two largest found in both maps. Selecting the seed locations based on the largest clusters (rather than statistical peaks) gave confidence that the majority of voxels in the seeds had the desired characteristic (e.g., condition-differences in a GLM), and is also consistent with findings of greater reliability from cluster-based statistical thresholds (e.g., Thirion et al., 2007). The seeds were located in the right inferior occipital gyrus, left inferior occipital gyrus, left fusiform gyrus, left superior temporal sulcus, right supramarginal gyrus and right postcentral sulcus (coordinates in Table 1). A 3-voxel radius sphere (with a volume of 123 voxels) was placed at each central voxel to create each seed.

**Table 1:** **Significantly connected regions for IC and FC analysis methods**.

**Region**	**Informationally connected clusters**				**Functionally connected clusters**			
	**Volume (voxels)**	***x***	***y***	***z***	**Volume (voxels)**	***x***	***y***	***z***
**UNIVARIATE SEED 1: RIGHT POSTCENTRAL SULCUS (*x* = 39, *y* = −42, *z* = 45)**								
Left precuneus	3871^*^	−11	−43	36				
Left fusiform gyrus	3871^*^	−30	−43	−10				
Left fusiform gyrus	3871^*^	−37	−58	−7	19	−33	−56	−18
Left middle temporal gyrus	3871^*^	−45	−58	19				
Left superior temporal gyrus	318^*^	−54	0	−6				
Left superior temporal gyrus	112	−54	−30	12				
Left parahippocampal gyrus	83^*^	−22	−12	−22				
Left temporal pole	83^*^	−19	8	−26				
Left anterior cingulate	78	−16	45	1				
Left inferior parietal lobe	3871^*^	−47	−43	47	31	−37	−38	53
Left orbital gyrus	318^*^	−35	27	−12				
Left inferior frontal gyrus	318^*^	−51	11	1				
Left middle frontal gyrus	87	−40	19	34				
Left superior frontal gyrus	3871^*^	−11	0	53				
Left superior frontal gyrus	3871^*^	−7	17	57				
Left caudate	170	−5	19	4				
Right inferior occipital gyrus	3871^*^	37	−67	−7				
Right fusiform gyrus	3871^*^	25	−78	−13				
Right fusiform gyrus	3871^*^	40	−36	−14				
Right superior temporal gyrus	3871^*^	39	−26	9				
Right precentral gyrus	3871^*^	55	2	22				
Right supplementary motor area	3871^*^	5	−19	54				
Right inferior frontal gyrus	3871^*^	46	33	6				
Right inferior frontal gyrus	3871^*^	34	7	31				
Right middle frontal gyrus	3871^*^	32	33	19	21	37	26	34
Right superior frontal gyrus	48	19	8	53				
Right cerebellum	39	44	−41	−44				
Right cerebellum	3871^*^	43	−43	−46				
Right cerebellum	3871^*^	23	−55	−45				
Right cerebellum	3871^*^	24	−43	−27				
Right thalamus	3871^*^	8	−15	1				
**UNIVARIATE SEED 2: RIGHT SUPRAMARGINAL GYRUS (*x* = 49, *y* = −24, *z* = 35)**								
Left lingual gyrus	96	−2	−79	4				
Left parahippocampal gyrus	59	−18	−25	−13				
Left middle temporal gyrus	29	−54	−56	19			
Left cingulate gyrus	306^*^	−12	7	30				
Left cingulate gyrus	911^*^	−2	−20	44				
Left supramarginal gyrus	911^*^	−60	−17	33				
Left precentral gyrus	911^*^	−29	−21	61				
Left inferior frontal gyrus	39	−30	23	−14				
Left cerebellum	89	−51	−56	−26				
Left cerebellum	66	−12	−68	−37				
Left thalamus	306^*^	−4	−10	15				
Right fusiform gyrus	37	37	−4	−29				
Right superior frontal gyrus	41	3	44	36				
Right cerebellum	87	30	−34	−26				
Right putamen	82	30	−11	−3				
Left supramarginal gyrus					12	−58	−26	23
Left precentral gyrus					10	−51	4	23
Left postcentral gyrus					27	−44	−30	42
Right postcentral gyrus					87	47	−8	16
**MULTI-VOXEL SEED 1: LEFT INFERIOR OCCIPITAL GYRUS (*x* = −30, *y* = −75, *z* = −7)**								
Left calcarine sulcus	225	−14	−96	−5			
Left fusiform gyrus	42	−33	−29	−23				
Left superior parietal lobe	28	−18	−63	51	13	−26	−60	42
Left orbital gyrus	22	−40	47	−5			
Left cerebellum	23	−37	−75	−37			
Left insula	22	−33	−7	16			
Right inferior occipital gyrus	66^*^	29	−85	−12			
Right middle occipital gyrus	66^*^	26	−86	12			
Right cerebellum	28	45	−67	−26			
Left middle occipital gyrus					40	−26	−60	−11
Left middle occipital gyrus					11	−33	−79	27
**MULTI-VOXEL SEED 2: LEFT SUPERIOR TEMPORAL SULCUS (*x* = −51, *y* = −41, *z* = 8)**								
Left calcarine gyrus	2401^*^	−15	−71	12			
Left fusiform gyrus	2401^*^	−45	−40	−22			
Left inferior temporal gyrus	2401^*^	−39	0	−26			
Left parahippocampal gyrus	43	−19	−8	−29			
Left superior parietal lobe	2401^*^	−30	−64	51			
Left postcentral gyrus	2401^*^	−27	−30	50			
Left inferior frontal gyrus	2401^*^	−53	14	2			
Right middle occipital gyrus	95^*^	33	−82	7			
Right lingual gyrus	2401^*^	16	−96	−7			
Right inferior temporal gyrus	95^*^	47	−59	−2			
Right angular gyrus	78	42	−70	38			
Right supramarginal gyrus	74	58	−41	38			
Right precentral gyrus	45	33	−23	57			
Right cerebellum	2401^*^	47	−59	−33			
Right cerebellum	2401^*^	12	−55	−15			
Right insula	146	35	−19	12				
**COMMON SEED 1: RIGHT INFERIOR OCCIPITAL GYRUS (*x* = 45, *y* = −61, *z* = −8)**								
Left fusiform gyrus	76	−44	−56	−14			
Left middle temporal gyrus	68	−54	−39	−5			
Left supramarginal gyrus	36	−65	−30	34			
Right precuneus	31	20	−48	36				
Right middle temporal gyrus	434	49	−72	12			
Right inferior parietal lobe	113^*^	43	−50	53			
Right supramarginal gyrus	113^*^	58	−41	38			
Right superior frontal gyrus	28	12	15	42			
Right superior frontal gyrus	62	16	53	1			
Right inferior occipital gyrus					97^*^	30	−84	−8
Right inferior temporal gyrus					97^*^	56	−53	−8
**COMMON SEED 2: LEFT FUSIFORM GYRUS (*x* = −38, *y* = −40, *z* = −16)**								
Left middle occipital gyrus	101	−41	−67	7			
Right middle occipital gyrus	101	51	−65	11			
Right supramarginal gyrus	49^*^	64	−40	29				
Right inferior parietal lobe	49^*^	53	−47	44			
Left superior occipital gyrus					12	−29	−71	26
Right fusiform gyrus					24	48	−54	−14

Significant regions are displayed for IC and FC (at p < 0.001 and cluster sizes determined by permutation testing). Similarly located regions are listed in the same row. Clusters significant at the seed's location are not listed to avoid circularity. Coordinates represent the peak of significant voxel–clusters. An asterisk indicates that the cluster contained multiple peaks, each included separately.

### Informational connectivity

The metric underlying IC quantifies how robustly the real class's activity pattern (versus the alternative classes) becomes discriminable at points along the timeseries. During correlation-based MVPA, the activity pattern at a time-point (i.e., a vector of voxel activations *m*-voxels long, recorded at that time) is compared to the mean voxel activity pattern corresponding to each condition in the held-out training set (i.e., the mean vector of each condition that is calculated by averaging the condition's time-points). We quantified multi-voxel pattern discriminability for each time-point with the following procedure (also captured in the formulae below):

1. Calculate the Pearson correlation coefficient between the (i) vector of voxel activation values for that time-point (i.e., its activity pattern) and (ii) vector of mean voxel activation values for the time-point's condition in the training data (i.e., the prototypical activity pattern for the condition). Fisher-transform to *z*-score.
2. Calculate the Pearson correlation coefficient between the (i) vector of voxel activation values for that time-point (i.e., its activity pattern) and (ii) vector of mean voxel activation values for each *alternate* condition in the training data (i.e., the prototypical activity patterns for the rival conditions).
3. Identify the highest correlation from step 2 (i.e., the highest similarity to an “incorrect” condition). Fisher-transform to *z*-score.
4. Multi-voxel Pattern Discriminability = Step 1 − Step 3 (i.e., Relationship to condition's prototypical pattern minus Relationship to the most similar incorrect condition).

The procedure is formalized in the below formulae, where $\bar{x}$ is the normalized 1-by-*m* row vector of *m* voxel activation values at time-point *n*, $\bar{y}$ is the normalized 1-by-*m* row training data vector of mean *m* voxel activation values for the correct (*c*) or incorrect (*i*) conditions relating to time-point *n*. In the analyses conducted here, *m* was 123 (the searchlight volume), and *n* ranged from 1 to 432. The artanh function normalizes the correlation coefficients through Fisher's transform.

$$\begin{array}{l} r_{c}[n]=\frac{\bar{x}[n]\cdot {\bar{y}}_{c}^{\prime }}{m-1}\\ r_{i}[n]=\max\left(\frac{\bar{x}[n]\cdot {\bar{y}}_{i}^{\prime }}{m-1},\;\forall i\neq c\right) \end{array}$$

Multi-voxel pattern discriminability = artanh(*r*_*c*_ [*n*]) − artanh (*r*_*i*_ [*n*]).

**Figure 2:**
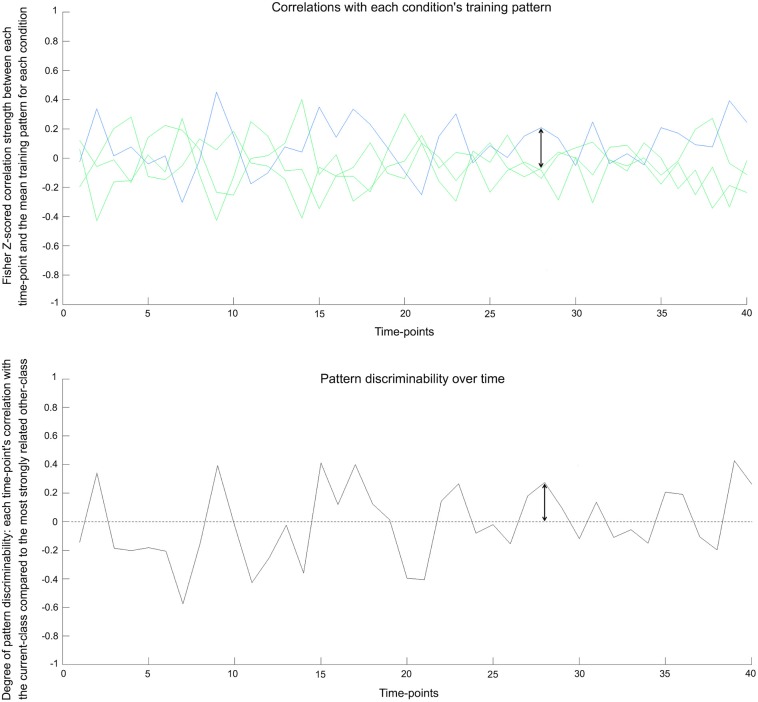
**Pattern discriminability over time in real data. Top:** The underlying basis for the pattern discriminability metric—shown here for the bottle condition in one seed in one subject. The blue line represents each time-point's Fisher *z*-scored correlation with the training pattern for the correct class. The green lines show the correlation values with mean training patterns for the three other classes. **Bottom:** Pattern discriminability is calculated by taking the correlation with the correct class's mean training pattern and subtracting the correlation strength of the strongest incorrect class (see text for details). When a time-point's value surpasses zero, it would reflect a classifier successfully predicting that time-point's condition. The arrow shows the corresponding values between the plots.

This multi-voxel pattern discriminability metric is calculated for each time-point across the timeseries, giving a dynamic series of values across the fMRI session (see Figure 2). This metric can be intuitively related to the typical binary metric used in classification analyses: The commonly used correlation-based classifier would successfully predict a time-point's condition when its data give a discriminability value above zero. This type of classifier makes a prediction for each time-point based on which class's training pattern is most strongly correlated with the time-point's activity pattern. In our measure, discriminability values are positive when a time-point's multi-voxel pattern is most strongly correlated with the training pattern of the correct class (i.e., the condition that was shown to participants). Positive discriminability values therefore reflect that a time-point's condition can be successfully predicted. A negative value on the other hand, reflects that the training pattern for a non-present (rival) class has the highest correlation with the current time-point, which would lead to an incorrect prediction.

To create an IC map, multi-voxel pattern discriminability is calculated for the timeseries of the seed region, followed by the timeseries of every searchlight sphere identified in the roaming searchlight procedure described above (Kriegeskorte et al., 2006). The timeseries of pattern discriminability from the seed region (i.e., a vector *N*-trials long) is then correlated with each searchlight's timeseries of discriminability, through a non-parametric Spearman's rank correlation. The resulting *r*_*s*_-value (representing the strength of the relationship between searchlight and seed) is placed at the voxel that lies at the center of each searchlight (a typical approach to mapping searchlight results; Kriegeskorte et al., 2006). This produces a brain map of values that each reflects how closely the timeseries of multi-voxel pattern discriminability for that (searchlight) area matches the equivalent timeseries of the seed region. The map therefore shows how strongly brain regions are correlated in terms of pattern discriminability (i.e., how “informationally connected” they are) with the ROI (the seed). Each participant's map is then Fisher-transformed into *z*-scores, spatially smoothed (8 mm FWHM) and tested for values above zero (i.e., asking which searchlights are significantly correlated with the seed) in a one-way group *t*-test. We have made the tools and scripts for running these analyses freely available within our IC Toolbox (http://www.informationalconnectivity.org). Statistical significance was tested using the same procedure (described below) for both IC and FC to enable direct comparisons.

### Functional connectivity

The IC results were compared to results from a typical FC analysis. We assessed FC for the same TRs analyzed using IC (TRs associated with the four man-made objects). The timeseries of mean activation values for the TRs was extracted for each seed region. This timeseries was then used as a predictor in a whole-brain GLM analysis, with six motion parameters and mean white matter signal as covariates. Individuals' maps of correlation values, reflecting the correspondence between voxels' and each seed's timeseries, were converted to Fisher-transformed *z*-scores and spatially smoothed (8 mm FWHM). All subjects' maps were subjected to a one-way group *t*-test for values greater than zero. The method for significance testing is outlined below.

### Significance testing

We adopted the same significance testing approach for both IC and FC to enable direct comparisons. For each seed region, the group statistical *t*-maps were first thresholded at *p* < 0.001 (and also at *p* < 0.005 to ensure that the results are not dependent on a particular *t*-threshold) for positive *t*-values in a one-way test to identify regions that were positively correlated with the seed. To correct for multiple-comparisons, we employed permutation testing to determine the minimum cluster size required for corrected significance. The seed's timeseries of values (pattern-discriminability values for IC; univariate activation values for FC) were shuffled by randomly swapping blocks of presentations (i.e., moving the sets of nine contiguous TRs that were separated by rest). One thousand group maps were created (constructed by randomly sampling from a set of 100 permuted maps for each subject) and submitted to a group test in the same manner as the seed's real (non-permuted) time-course, including thresholding at *p* < 0.001. This gave a null distribution of 1000 group maps. We used this to determine the minimum cluster size needed in the real (non-permuted) group map for a corrected *p*-value of < 0.05: The size of the largest cluster within each permuted group map was extracted, giving 1000 cluster sizes. The 50th largest cluster size from this null distribution is the cluster size that would be expected by chance five times out of 100 (i.e., *p* < 0.05). Any clusters larger than this in the true (non-permuted) group map are significant at *p* < 0.05 corrected. This approach has the advantage of correcting in a manner that accounts for the dataset's own level of smoothing, as each permutation underwent the same processing as the true order. The minimum cluster sizes were calculated separately for every seed and the two connectivity approaches.

## Results

We analyzed a dataset collected while subjects viewed blocks of images of four types of man-made objects, using our novel IC method to track and compare dynamic change in discriminability of multi-voxel patterns across time. We compared these results to a typical FC analysis that tracks synchronized changes in univariate activation. We employed six seeds, selected from regions showing univariate variation between conditions, MVPA decoding, or both.

**Figure 3:**
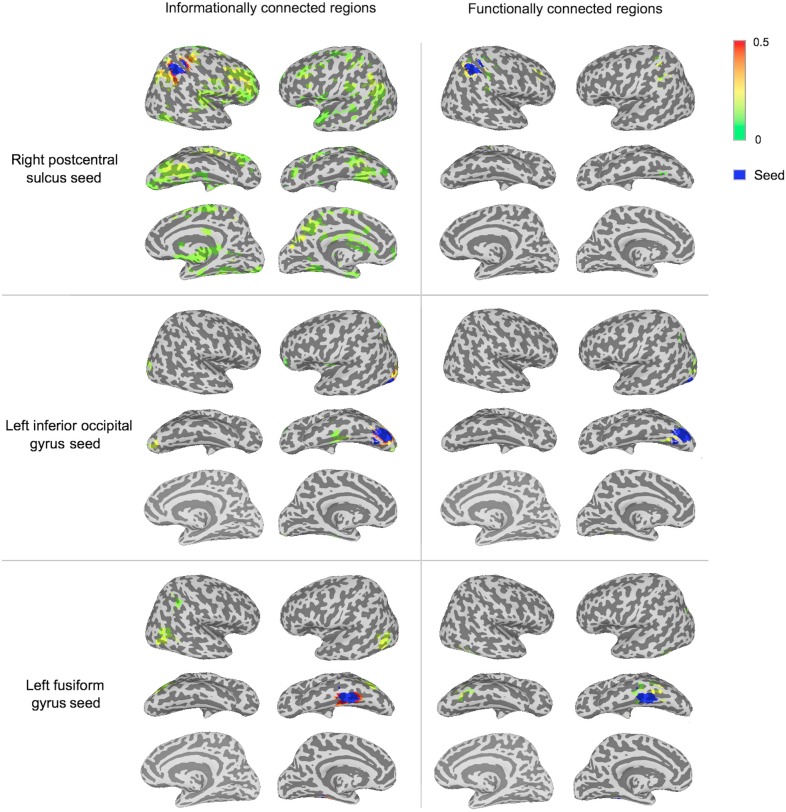
**Significantly connected regions in IC and FC analyses for three of the seeds.** A group *t*-test (*p* < 0.001 with minimum cluster size from permutation testing) determined significance (described in the “Materials and Methods”). Connectivity strength is displayed between green (lower values) and red (higher values). Each seeds region is shown in blue.

The IC and FC analyses identified different networks of regions, with IC revealing larger networks than FC in this man-made object dataset (Figures 3, 5; Table 1). These FC results were not specific to the *p*-value selected: Repeating the FC analysis with a more liberal *p*-value (*p* < 0.005 with a permutation-generated minimum cluster size) generated similar networks of regions. The different seeds varied in how many regions were informationally connected to them: for example, the right postcentral sulcus seed was informationally connected with a large variety of cortical areas, while the left inferior occipital gyrus seed was not (Figure 3).

**Figure 4:**
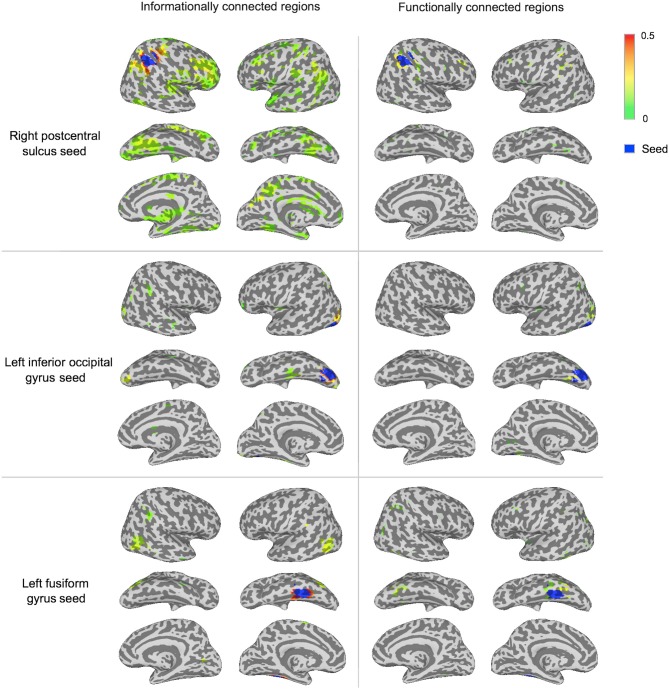
**Connectivity strengths before cluster-based thresholding for three of the seeds.** The displayed regions have connectivity above zero from the group *t*-test at *p* < 0.001 prior to thresholding in cluster-based permutation tests, to visualize sub-threshold connectivity for both methods. Connectivity strength is displayed between green (lower values) and red (higher values). Each seed region is shown in blue.

To visualize the two methods' results without a minimum spatial extent, Figure 4 shows IC and FC connectivity before applying the cluster-based permutation thresholds.

**Figure 5:**
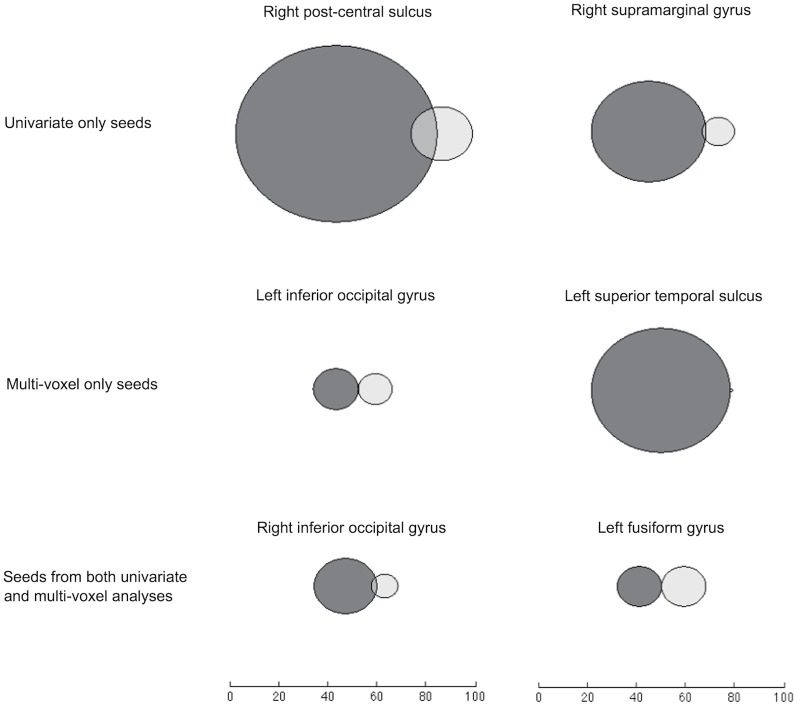
**Venn diagrams of voxels significantly connected to each seed through IC (dark gray) and FC (light gray).** Searchlights that overlapped with the relevant seed region have been removed. Here, FC results come from an analysis using the timeseries of *searchlights'* (rather than voxels') mean values, to give a suitable comparison with the searchlight-based IC results.

By visualizing the degree of overlap in regions that were significantly informationally and functionally connected with each seed, we found that the two methods identified either largely distinct or slightly overlapping networks of regions (Figure 5). This is also reflected in the small number of regions that are listed under both methods in Table 1.

Many of the areas showing synchronous multi-voxel pattern discriminability include regions that have been implicated in object processing. Evidence underlying this involvement is presented in the discussion.

**Figure 6:**
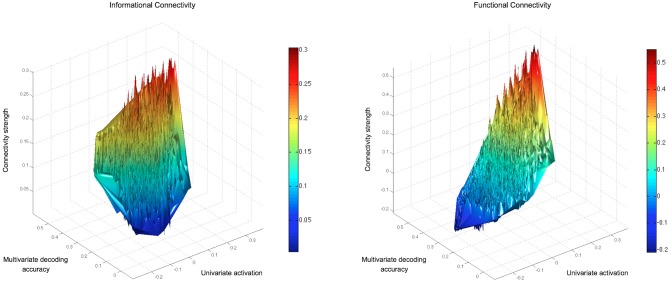
**Connectivity strengths of all searchlights with a seed in the left fusiform gyrus (present in both the GLM and MVPA searchlight results).** The IC and FC results for every brain searchlight are displayed relative to the searchlight's mean univariate activation to the objects and decoding accuracy in a 4-way classification of object-types. Searchlights that overlapped with the seed region have been removed. The FC values reflect the described FC approach, using each searchlight's mean timeseries (rather than each voxel's timeseries) to give a suitable comparison with IC (which reflects information in a searchlight volume). The empty space visible in the top-left octant of the FC graph for searchlights with low response levels (despite high decoding accuracy) highlights connectivity that is inaccessible to univariate FC.

We examined the univariate and multivariate characteristics of searchlights, relative to their levels of IC and FC with seeds, and confirmed that the IC approach can highlight regions that would otherwise be ignored by FC. For example, regions with low univariate activation to conditions, yet decodable multi-voxel information, were ignored by FC, but detected with IC. This can be seen in Figure 6, which shows the group average mean activation, multivariate information and connectivity strength (with the left fusiform gyrus seed) of searchlights across the brain. The empty space visible in the top-left octant (representing searchlights with low response levels despite high decoding accuracy) in the FC, but not IC, graph highlights the connectivity that is inaccessible to univariate FC. This pattern was representative of connectivity with other seeds.

**Figure 7:**
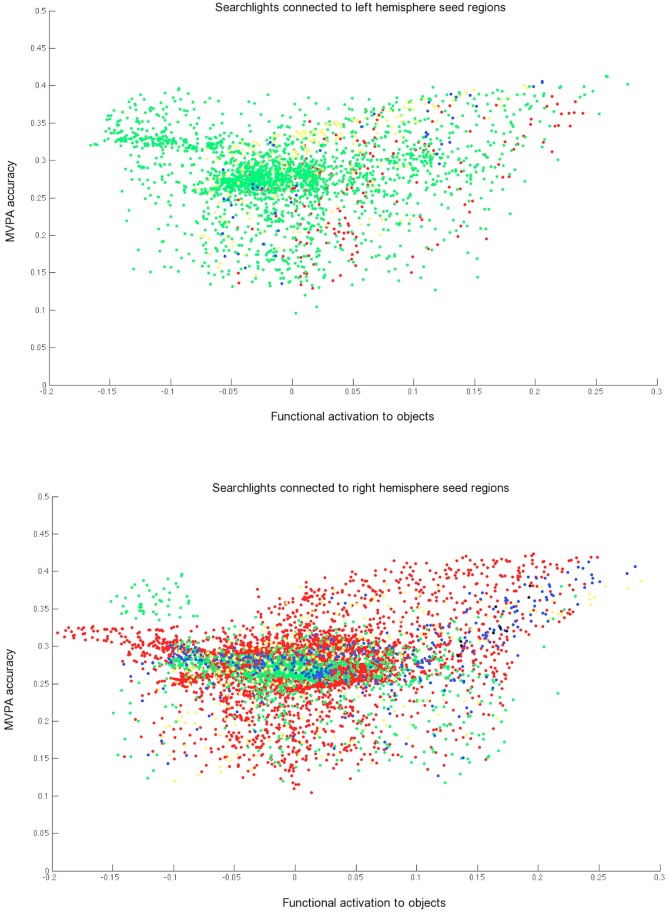
**Searchlights with significant informational connectivity to at least one of the three left hemisphere seeds (top) and at least one of the three right hemisphere seeds (bottom), shown against MVPA accuracy and mean functional activation.** The green, yellow, and red colors each represent searchlights that are connected with just one seed. Blue points show searchlights that are connected to two seeds and black points show searchlights connected to three seeds. Searchlights overlapping with one of the three seeds regions were removed from each scatterplot.

The IC networks detected for each seed were not redundant with each other. A large proportion of searchlights were significantly connected with only one seed (Figure 7) and although some searchlights were identified in the networks of two seeds (blue in Figure 7), very few were found for three. The distinctiveness apparent for different seed networks also confirms that IC is not redundant with conducting a typical MVPA searchlight analysis, as it can highlight distinct networks based on the selected seed.

## Discussion

This paper has presented a new method—IC—for measuring synchronous discriminability of multi-voxel patterns across the brain. We have described a metric for quantifying multi-voxel pattern discriminability across a timeseries, and conducted an IC analysis on data collected as subjects viewed four types of man-made objects. The IC method identified networks of synchronized regions that were not identified by FC. Many of these brain areas are linked to object processing (discussed below), suggesting that multi-voxel pattern discriminability can identify networks involved in processing conditions that are characterized by multi-voxel information (such as perceiving objects).

The limited overlap of regions identified by IC and FC is consistent with a prior report of low commonality between MVPA and univariate measures, with MVPA having greater sensitivity overall (Jimura and Poldrack, 2012). GLM and MVPA approaches have been conceptualized as tapping basic processing (causing changes in univariate activation) versus decoding representations of the content being processed (causing changes in pattern discrimination; Mur et al., 2009; Jimura and Poldrack, 2012; although MVPA has also been applied to identify cognitive processes e.g., Esterman et al., 2009). MVPA investigations into representational content, such as the type of man-made object being processed, have proven effective for advancing our understanding of the visual system (Eger et al., 2008) and others (e.g., auditory system: Lee et al., 2011). Analogously, identifying networks characterized by synchronized discriminability of multi-voxel information will be valuable for investigators wishing to study how systems of brain areas are engaged. A related proposed distinction between MVPA and GLM, which frames MVPA as reflecting *sub*-processing that varies during GLM-measured general processing (Jimura and Poldrack, 2012) suggests that IC's access to multi-voxel patterns would be valuable for mapping sub-processing networks.

Although a comprehensive discussion of implications of specific findings from this analysis, in terms of our understanding of the visual system, is beyond the scope of this paper, we will make some comments on the types of hypotheses that can be informed by this approach. Firstly, the IC findings are consistent with theories that an object's action representations become automatically activated when its visual or semantic properties are engaged (Chao and Martin, 2000; Johnson-Frey, 2004; Mahon and Caramazza, 2009). A frontal region, the left inferior frontal gyrus, has previously been linked to visual-to-motor transformations (Chao and Martin, 2000) and was informationally connected to several of the seeds here. Equally, the supramarginal gyrus, suggested as a location for representations of object-use skills (Johnson-Frey, 2004), was informationally connected to four of the seeds. Secondly, the distinctions between the IC and FC results for the left fusiform gyrus seed are consistent with a prior fMRI investigation into the organization of object-processing regions (Mahon et al., 2007). Mahon and colleagues (2007) have reported that while the left and right fusiform gyri respond similarly to different object categories in terms of their mean BOLD activation, their underlying neural representations (when measured through repetition suppression) differ. This is supported by the IC and FC differences reported here: the left and right fusiform gyri were *functionally* connected (fitting with Mahon et al.'s mean activation findings) but not *informationally* connected (for the same statistical thresholds), giving support for the left and right fusiform regions containing differences in their object representations (Mahon et al., 2007). This study is the first to find that object-processing regions are linked together by common fluctuations in multi-voxel patterns for different types of man-made objects.

As a primary analysis method, a key advantage of IC is its ability to examine synchrony within condition-related information that is not accessible from univariate response levels, such as object identity. Dynamically changing cognitive states (such as attention to objects or visual properties) will also differentially affect systems during the time-course of an experiment. For example, time-points marked by greater or reduced attention will likely show increased or decreased pattern discriminability. Regions that process stimuli as part of an interconnected system will often share these effects. As well as acting as a primary analysis method, IC can be used as a further analysis after an MVPA searchlight procedure, which is often used to identify regions that have condition-relevant information or a relationship to individual differences (e.g., Coutanche et al., 2011). The brain regions identified in a searchlight analysis will likely decode conditions using a variety of separation principles and forms of relevant information (i.e., the analysis is “opportunistic”; p. 550, Jimura and Poldrack, 2012). For example, man-made objects could be separated by visual appearance in early visual areas, viewpoint-independent identity in later visual areas, associated motor movements in motor areas, and so on. A region's basis for its distributed information will strongly influence which stimuli and time-points are particularly discriminable. The IC approach can help identify different networks of regions, moving beyond one overall MVPA searchlight map. The ability to separate regions based on decoding principles is visible in the IC results for a left inferior occipital gyrus seed in this work. This posterior occipital region showed strong IC with occipital regions in the opposite hemisphere, but little other contralateral cortex. In contrast, more anterior seeds had more extensive IC. This result was expected, given the basic visual properties that are processed in these early visual areas (Kamitani and Tong, 2005). Once the visual processing stream moves to more anterior brain areas, the processing target moves away from basic visual properties to whole objects, which are processed across different brain regions.

Among other applications, IC can also be used to compare groups by directly contrasting subjects' IC values, or to examine differences in IC strengths between tasks. For example, certain networks may show connectivity increases if participants make action-related, compared to visual, judgments of objects. The IC method's general framework can be extended to use classifiers other than the correlation-based approach employed here. Many classifiers, including support vector machines, assign continuous values to the potential conditions for each time-point. These condition-weights determine a classifier's predictions, and incorporate how well the conditions' multi-voxel patterns can be distinguished from each other. By extracting and treating these values in the manner outlined here for correlations (i.e., correlating a timeseries of classifier condition-weights instead of *z*-scored correlation coefficients), investigators can draw on the advantages of a range of classification methods.

Although we employed IC using spherical volumes for seeds and searchlights, the method is compatible with seeds and targets that are defined in other ways, such as through anatomical masks or a separate functional localizer. In some cases, it might be desirable to select a seed with a theoretically driven size. For example, an investigator may wish to ensure that the entire visual field of retinotopic V1 is selected as a seed so that the pattern discriminability metric reflects the information available from this entire region. Future investigations that employ both FC and IC can examine seeds that are defined according to a variety of criteria. Here we selected the univariate-based seeds using GLM contrasts—as this was directly comparable to the multivariate-seeds, in which the conditions' multi-voxel patterns were separable—but a connectivity seed can be defined in a number of different ways, such as selecting regions with high within-condition variance. The seed and data used in an FC or IC analysis may be influenced by the particular question under investigation. Whereas studies of the object-processing system, for example, may examine a timeseries that fluctuates with different conditions, other targets, such as the influence of attention, may be accessible from seeds that show fluctuating responses within a condition.

One methodological question concerns the length of a time-series required for robust IC results. The specific data requirements for a given experiment will depend strongly on a number of factors, including the conditions that trigger the data. For IC, experimental paradigms that extensively sample a stimulus space, or that challenge a neural system to varying degrees, will likely produce strongly fluctuating multi-voxel discriminability, potentially increasing the opportunity to sensitively detect relationships between regions. Similarly, an engaging task will likely reduce participant fatigue, and more reliably engage neural representations, thereby producing a more robust measure of discriminability at each time-point. In addition to influencing the quantity of time-points in the IC timeseries, the amount of collected data will influence the robustness of the training model. This factor is well known to MVPA investigators, and readers are referred to relevant discussions (e.g., O'Toole et al., 2007; Mur et al., 2009) or approaches to improving training data (e.g., Coutanche and Thompson-Schill, 2012) for further information. We note that for the data analyzed here, we observed (from re-running analyses with randomly selected subsets of runs) that the reported informationally connected regions reached significance (as measured with a group mean *t*-value) when the subjects' IC values were calculated from a minimum of between seven and 11 runs (depending on the seed). For this particular set of stimuli and participants, approximately 7–11 blocks of each condition were therefore sufficient for identifying the brain networks reported above.

Although we found that a prototypical FC analysis was unable to identify the networks found using IC, we acknowledge that a variety of FC analysis measures are available, and others may be more effective. Future work may wish to compare IC results to other FC analysis approaches. Equally, there may be circumstances where investigators wish to track variations in a general process, without influence from sub-process or representational nuances. Analyzing data with FC or IC does not preclude using the other method: in many circumstances, they could be used together and their results compared, as discussed above for the left and right fusiform gyri. A joint approach may lead to a more nuanced understanding of relevant networks.

We have created and made available an IC Toolbox online (http://www.informationalconnectivity.org) to aid investigators in applying this technique to their own data.

### Conflict of interest statement

The authors declare that the research was conducted in the absence of any commercial or financial relationships that could be construed as a potential conflict of interest.
